# Child-Patient Perspective on Results After Correction of Sagittal Synostosis and the Difference Between Child-Patient and Parent’s Perspectives

**DOI:** 10.1097/SCS.0000000000010263

**Published:** 2024-05-09

**Authors:** Melissa S.I.C Kurniawan, Nathalie W. Kamst, Irene M.J. Mathijssen, Nicole S. Erler, Marie-Lise C. van Veelen

**Affiliations:** * Department of Plastic and Reconstructive Surgery and Hand Surgery, Erasmus University Medical Center Rotterdam; † Department of Neurosurgery, Erasmus University Medical Center Rotterdam; ‡ Department of Biostatistics, Erasmus University Medical Center Rotterdam; § Department of Epidemiology, Erasmus University Medical Center Rotterdam, The Netherlands

**Keywords:** Esthetic outcome, craniosynostosis, patient-reported outcome, sagittal synostosis

## Abstract

**Objective:**

This study assesses the level of child-patient satisfaction with the surgical result after scaphocephaly correction and the difference between child-patient and parents' perspectives.

**Methods:**

A questionnaire was sent out to children between 6 and 18 years old with isolated sagittal synostosis, who had undergone either a frontobiparietal remodeling or extended strip craniotomy, and separately to their parents.

**Results:**

The questionnaire was completed by 96 patients, 81.2% of the patients considered their head to be similar or slightly different from others. Despite the majority being satisfied with the outcome, 33% would change the shape of their head if they could. Patients who underwent extended strip craniotomy wanted to change the back of their head more often (*P* = 0.002), whereas patients who underwent frontobiparietal remodeling wanted to change their forehead (*P* = 0.005). The patients’ own perspective on head shape was independent of the cephalic index (CI). However, patients with a relatively narrow CI received more remarks from others about their heads (*P* = 0.038). Parent and child agreement was 49.7% on average. Differences between child-patients and parents were found in reporting adaptive behavior.

**Conclusion:**

The majority of patients were satisfied with the outcome of their intervention. The child’s perspective seems to be a valuable addition to evaluate sagittal synostosis surgery as it is independent of the CI and differentiates between different surgical techniques. In addition, the patient’s perspective is comparable to the parent’s perspective, but gives more details on adaptive behavior.

Scaphocephaly is a type of craniosynostosis characterized by the premature fusion of the sagittal suture. This restricts skull growth perpendicular to the sagittal suture and increases longitudinal growth; this results in a long and narrow skull.^1^

Besides a deformed skull, scaphocephalic growth restriction can also lead to increased intracranial pressure, which may cause visual impairment and headaches. Neurodevelopmental deficits, such as learning and attention problems, can be found in large cohorts of patients^1–5^; their relationship to skull deformity, intracranial pressure, and the extent and timing of surgery, remains a topic of study.

Skull remodeling surgery is performed in young patients with the aim to prevent these problems from occurring and improve esthetics. Various surgical techniques are available to correct sagittal suture synostosis, ranging from invasive techniques, such as total cranial vault remodeling, to minimally invasive techniques, such as spring-assisted correction.

Outcome parameters including occipital-frontal circumference, cephalic index (CI), skull volume, the presence of papilledema on fundoscopy, and optical coherence tomography are measured during follow-up visits up until the age of 18.

However, it can be difficult to objectively assess cosmetic end results; which is one of the key goals of craniofacial surgery.^6^ Several cosmetic scoring systems exist, with the one by Whitaker et al^7^ being the most commonly used. This score is determined by the surgeon and is used to determine whether additional surgery is necessary. Although the Whitaker scoring system can be used for all craniofacial deformations, specific traits cannot be rated, and it does not take the patient’s own opinion into account.

One of the current methods for evaluating the cosmetic end results after scaphocephaly correction is based on photographic scores. Observers rate postoperative photographs based on 5 characteristics on a 3 to 5-point scale.^8,9^ However, these scores do not incorporate the perspective of the patients themselves. Therefore, the currently available measurement tools for evaluating cosmetic outcomes in scaphocephaly correction are somewhat limited.

Previous studies concerning cosmetic end results have relied on expert opinions,^9–13^ parent perspectives,^14–16^ or a combination of both.^17,18^ However, the patient’s perspective on the esthetic has been lacking, even though this is essential and helpful information.

The aim of the study is to assess the level of satisfaction among child-patients who have undergone scaphocephaly correction surgery in terms of both esthetics and functional outcomes. The questionnaire will be correlated to the age at the time of questionnaire completion, the CI, and the type of surgery. A secondary aim of the study is to compare the outcomes reported by child-patients with those reported by their parents.

## METHODS

The questionnaire used in this study consists of 9 questions, with 5 questions focused on esthetics, 2 on functional outcome, and 2 open-ended questions about the content of the questionnaire itself (Appendix A, Supplemental Digital Content 1, http://links.lww.com/SCS/G199). The questionnaire was designed in consultation with a panel of 10 patients and their caregivers, as well as physicians and a psychologist from the craniofacial team. It also includes a limited number of questions based on published recommendations.^19^ All responses were rated on a 5-point Likert Scale.

The questionnaire was distributed to children aged 6 to 18 years who had been operated on for computed tomography–proven, isolated sagittal synostosis in the Dutch Craniofacial Centre (Sophia Children’s Hospital, Rotterdam) between 1990 and 2008. During this time period, there was a change in surgical protocol at the hospital. In 2005, the extended strip craniotomy (ESC) technique was introduced and performed in children presenting with sagittal synostosis before the age of 6 months. After the age of 6 months, patients underwent an frontobiparietal remodeling (FBR). The location of the scar differs between the two techniques, with the skin incision for the ESC running from above one ear across the occiput to the other ear,^20^ and the incision for a FBR consisting of a bicoronal zigzag.^21^

Patients who had syndromic sagittal synostosis or multiple closed sutures were excluded from the study. Included patients were asked to complete the questionnaire based on their own interpretation and perspective. The study protocol was approved by the Institutional Human Research Ethics Board (Erasmus University Medical Center: MEC-2014–445) and followed the statements of the Declaration of Helsinki. The instructions for the questionnaire specifically indicated that patients should answer the questions themselves to the best of their ability. If they were not able to read well enough to complete the questionnaire by themselves, they were allowed to ask a parent for help. After a period of 12 months, the parents of the participating children were asked to complete the same questionnaire, without consulting their child. This timeframe was used to minimize the possibility of patients and parents influencing each other’s responses.

Patients were asked to complete the questionnaire solely on their own perspectives and opinions. Data on complications and reinterventions were collected from patients’ medical records, and data on cranial width and length were measured from skull x-rays. The CI was calculated by dividing cranial width by length, with the mean CI being determined using the mean of the 3 most recent values. If fewer than 3 postoperative skull x-rays were available, mean CI was calculated using the available data.

The severity of the preoperative scaphocephalic shape may influence the parents’ long-term opinion of the results. The magnitude of difference between pre and postoperative CI is, therefore, used as a separate measurement, when available.

### Statistical Analyses

Patient characteristics and responses to the questionnaire are presented with counts and percentages (for categorical variables), means and SDs (for approximately normally distributed variables), or with median and interquartile range (IQR; for non-normally distributed continuous variables). The χ^2^ test was used to test for differences among patients who received different surgical techniques.

The relation between the answers and age at questionnaire completion was evaluated using the Spearman correlation coefficient and test. Moreover, Spearman correlation coefficients and tests were calculated to test correlations between adaptive behavior and comparison of head shape, remarks from others, and visibility of the scar, respectively.

The Kruskal-Wallis test was used to determine whether the mean CI was related to the patient’s own perspective on their head shape and/or the opinion of others, for example, thus the patient’s own opinion, and the number of remarks made by others. χ^2^ test was used to explore the relation between the surgical techniques used and the desire to change a specific part of the head.

To assess the agreement between the responses of parents and patients, the Cohen Kappa interrater reliability and the percentage agreement were calculated. A one-point difference between parents and patients on the Likert Scale was defined as an acceptable difference in agreement.

Answers were excluded from the analysis when multiple answer boxes were ticked, with the exception of the question about which part of the head the patient would like to change. All analyses were performed with the R version 3.6.2.^22^.

## RESULTS

A total of 145 patients met the inclusion criteria and were sent a questionnaire; of which 96 patients (66.2%) returned completed questionnaires for analysis. The attrition analysis revealed no significant differences between the responders (n = 96) and nonresponders (n = 49) in sex, type of surgery, age, and pre and postoperative CI (Supplemental Digital Content, Table 1, http://links.lww.com/SCS/G200). Of the 96 included patients, 45 patients had undergone an FBR and 51 patients had undergone an ESC. The characteristics of the patients are presented in Supplemental Table (Supplemental Digital Content, Table 2, http://links.lww.com/SCS/G200); the majority of respondents were males (77.8%). The median age at the time of completing the patient questionnaire was 11.6 years (IQR: 9.0–13.0) and the mean CI was 72.3 (SD: 4.26). Three patients had missing CI data due to a lack of skull x-rays during follow-up. The age at surgery and age at completing the questionnaire were significantly different between the patients undergone FBR and patients undergone ESC due to the change in surgical protocol. There were no differences in complications or reintervention rates between surgical techniques.

Answers to the questionnaire are presented in Supplemental Table (Supplemental Digital Content, Table 3, http://links.lww.com/SCS/G200). For all questions focusing on esthetics, there were no significant differences between the surgical techniques. Overall, 81.2% of the patients considered their head shape to be (completely) similar or slightly different from others (question 1, Appendix, Supplemental Digital Content 1, http://links.lww.com/SCS/G199).

Of the patients, 60% reported the scar to be barely noticeable/unnoticeable (question 2, Appendix, Supplemental Digital Content 1, http://links.lww.com/SCS/G199). Of the patients, 75.0% never received remarks from others about the shape of their heads (question 3), and 37.9% of the patients never considered the shape of their heads when making decisions (question 4). This question explored adaptive behavior (eg, deciding on a hairstyle, or going for a swim and having wet hair). Most respondents (66.7%) did not want to change any part of their head (question 5). A total of 34 patients (11 FBRs; 21 ESCs) wanted to change at least one item on their head (Supplemental Digital Content, Table 4, http://links.lww.com/SCS/G200). Significantly more patients wanted to change their forehead if they had a FBR (45.45%) compared with ESC (4.8%). In contrast, patients who received an ESC were more likely to be less satisfied about the back of their head (66.7%) compared with those who received a FBR (9.09%).

A large proportion (29.1%) of patients reported experiencing headaches, ranging from “monthly” to “daily” (question 6). Patients who received an ESC reported significantly more frequent than those who received an FBR. Specifically, 25.5% of the patients who underwent ESC reported weekly headaches compared with 4.4% of the patients who underwent FBR. In addition, 40.5% of the respondents reported being often or regularly distracted (question 7).

The visibility of the scar and frequency of headaches had a significant correlation with age at completion (Supplemental Digital Content, Table 5, http://links.lww.com/SCS/G200). However, these correlations were weak. None of the other questions had a correlation with age at completion. In addition, it was observed that there was a mild correlation between adaptive behavior and the patient’s own perception of the head shape, and remarks from others (Supplemental Digital Content, Table 6, http://links.lww.com/SCS/G200).

**FIGURE 1:**
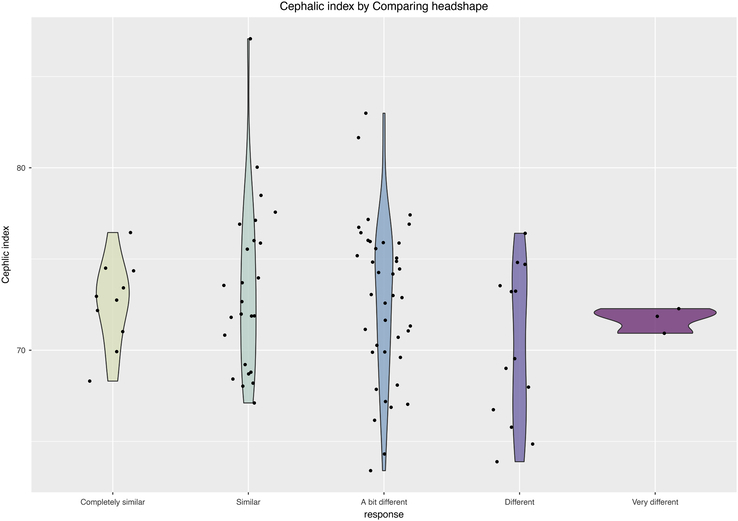
Mean CI per answer category for the question “Others make remarks about the shape of my head…” (*P* = 0.038; Kruskal-Wallis test). CI indicate Cranial Index.

**FIGURE 2:**
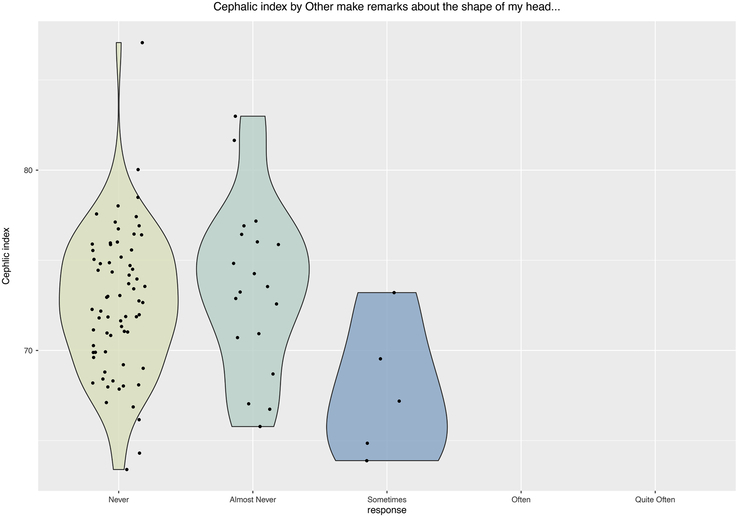
Mean CI per answer category for the question “When I compare the shape of my head to that of others. I find it…” (*P* = 0.400; Kruskal-Wallis test). CI indicate Cranial Index.

One might assume that the severity of the scaphocephalic shape may influence the perspective of the patient, but also the perspective of the environment. The study found that there is no relationship between CI and the patients’ perception of how different their head shape is compared with the head shape of others (question 1, *P* = 0.400; Fig. 1). However, patients with a relatively small CI reported significantly more remarks from other people about the shape of their head (question 3, *P* = 0.038; Fig. 2).

Preoperative CI was available in 49 patients. From patients’ and parents’ perspectives, the magnitude of change in preoperative and postoperative CI was not correlated to any of the questions (Supplemental Digital Content, Table 7, http://links.lww.com/SCS/G200).

### Parents and Patients Agreement

**FIGURE 3:**
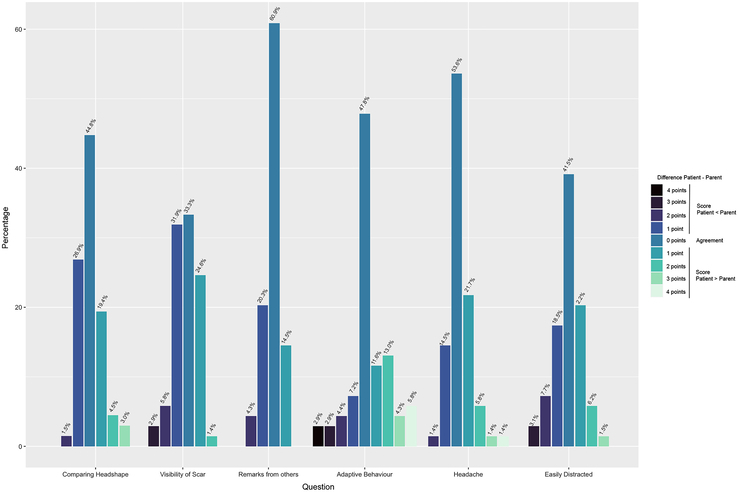
Difference in score per question between patients and parents.

In total, 69 parents completed the same questionnaire as their children. Interrater reliability showed a fair to moderate agreement between parent and child in the majority of the questionnaire items, with an average agreement of 49.7% (Supplemental Digital Content, Table 8, http://links.lww.com/SCS/G200). In 17.4% of the cases, patients rated one point higher on the Likert Scale than the parent and 17.9% one point lower (Fig. 3). The question regarding adaptive behavior (question 4) showed that 16 patients (23.2%) rated the question at least 2 points higher compared with the parents. This indicates that patients are more likely to adapt their behavior due to the shape of their heads or the scar (eg, deciding on a hairstyle, or going for a swim and having wet hair), without their parents noticing this adaptive behavior. The agreement between parents and patients did not change based on the age of the patient when completing the questionnaire (Supplemental Digital Content, Table 9, http://links.lww.com/SCS/G200)

## DISCUSSION

The study investigated the patient’s own opinion on their appearance and function after surgical correction. This study also looked at the patients’ perspectives on the overall appearance and specific aspects of their heads and how others react to them, as well as the agreement between patients and their parents.

The results indicate that the majority of the patients are satisfied with their appearance, regardless of their type of surgery. However, more than one-third reported adjusting their behavior due to their head shape and/or scar, such as a specific hairstyle to hide certain aspects of their head.

The study found that patients who had a FBR had a higher tendency to be less satisfied with their forehead. In contrast, patients who had an ESC wanted to change the shape of the back of their head. This is noteworthy because the correction of the forehead is included in the FBR procedure but not in the ESC procedure. This may be due to temporal pinching or to irregularities of the forehead after remodeling in FBR. It may also relate to the spontaneous normalization of the bulging forehead in ESC. Concerning the back of the head, radial incisions of the occipital bone are included in the FBR but not in the ESC. At the time of this writing, the CI was the most frequently used parameter to describe postoperative esthetic outcomes. The CI is related to the environment’s perception on the patients’ head, whereas the CI is not correlated with patients’ perception of how different their head shape is compared with others. The questionnaire and the CI are, therefore, complementary parameters. The combination can be used to evaluate outcomes during follow-up.

In our study population, the prevalence of patients with frequent headache complaints is 30.2%. Literature shows a variation in the prevalence of headaches in the general population.^23–28^ A Dutch population-based cohort study,^29^ showed that 23% of the children in their cohort experience headaches “sometimes” to “often.” Due to a higher prevalence in patients with sagittal synostosis compared with the general population, a follow-up study involving a detailed questionnaire on headache complaints was conducted in these patients. The results of this study are discussed by van de Beeten et al (2019).^30^

To gather the patients’ perspectives on their esthetic outcomes, the study included patients who were old enough to understand and answer the questions themselves. Therefore, the present study focused on patients who had undergone FBR or ESC between 1990 and 2008. This resulted in a sample of patients who underwent surgery in a more distant past, using techniques and measurements that may be less used in current practice. For example, the FBR technique and the zigzag incision are still used in modern practice, whereas the ESC technique is becoming less frequently used. It is noted that newer, minimally invasive surgical techniques with smaller scars have been developed^31–33^; this questionnaire could be used to evaluate esthetic outcomes after the newer types of procedures.

Most questionnaires have been developed for the parents to fill out; our study found that young patients themselves can have a meaningful assessment of the surgical result. Agreement between patients and parents was seen in 49.7% of the cases. In 35.3% parents and patients differed only by 1 point on the Likert scale, for example, “unnoticeable” and “barely noticeable.” However, conclusions from these results must be taken carefully as younger patients were allowed to ask their parents for help. In 23.2% of the patients scored higher than their parents on adaptive behavior, indicating that patients were more conscious about the shape of their head and were actively covering their head or adapting their behavior, whereas the parent is less aware of this problem/behavior. Thus, the patient’s perspective, therefore, offers additional value to the parent’s perspective.

There are 3 limitations that must be taken into account when interpreting the results.

First of all, the use of a self-administered survey as well as a small sample size, raises the question of possible selection bias. The attrition analysis shows no differences in baseline characteristics between responders and nonresponders, which minimizes the risk of selection bias. With the response rate in our study, we adhere to the minimum required response rate of 60% by some journals.^34^ However, our study is still based on a fraction of the total population. To increase the reliability and validity of the results, the study must be repeated in a larger study population.

Second of all, if patients are not able to complete the questionnaire by themselves, they are advised to ask a parent or guardian for help. Especially the younger patients who might have asked for help. In the instruction, investigators have emphasized that the patients' questionnaire should solely be based on the patient’s perspective. However, when completing the questionnaire with the help of a parent/guardian, there is a possibility that the patient’s perspective is influenced by their parent/guardian. This can lead to biased data. However, the study population who completed the questionnaire has a median age of 11 years (IQR: 9.0–13.0 y). The majority of the patients were able to read well enough to complete the questionnaire by themselves. In addition, young patients are more likely to ask a parent for help. If this were the case in our study, the agreement between patients and parents would have been higher in younger patients. However, the study did not find a correlation between the age at completing the questionnaire and agreement between patients and parents. This would suggest minimal bias in the data.

Finally, our study used a questionnaire custom-designed for this research, making it a nonvalidated instrument. Existing and validated questionnaires lacked craniosynostosis-specific items and were, therefore, not considered appropriate tools for our study. At the time of this writing, the FACE-Q is in development for craniofacial anomalies.^35^ This validated questionnaire will primarily focus on evaluating satisfaction with facial characteristics from the patient’s perspective, and less on the shape of the skull.^36^

This study has highlighted the importance of child-patient assessment and the inclusion of questions about head shape. To increase its utility for these patients, it would be beneficial to expand the FACE-Q to include questions about the shape of the skull, in addition to those about the forehead.

## CONCLUSIONS

In this group of patients aged 6 to 18, who underwent surgery for isolated sagittal synostosis, the majority reported being satisfied with the results of their surgery. The patients’ perspective provides valuable information beyond current outcome parameters, as it is independent of the CI and enables differentiation between surgical techniques. Furthermore, the patients’ perspective is comparable to the parents’ perspective. However, patients have a tendency to be more conscious about their head shape and show adaptive behavior, whereas the parents are less aware of this problem/behavior.

## Supplementary Material

SUPPLEMENTARY MATERIAL

## References

[1] Renier D Lajeunie E Arnaud E . Management of craniosynostoses. Childs Nerv Syst 2000;16:645–658 11151714 10.1007/s003810000320

[2] Alperovich M Runyan CM Gabrick KS . Long-term neurocognitive outcomes of spring-assisted surgery versus cranial vault remodeling for sagittal synostosis. Plast Reconstr Surg 2021;147:661–671 33620934 10.1097/PRS.0000000000007640

[3] Kapp-Simon KA Speltz ML Cunningham ML . Neurodevelopment of children with single suture craniosynostosis: a review. Childs Nerv Syst 2007;23:269–281 17186250 10.1007/s00381-006-0251-z

[4] Kljajic M Maltese G Tarnow P . The cognitive profile of children with nonsyndromic craniosynostosis. Plast Reconstr Surg 2019;143:1037e–1052ee 10.1097/PRS.0000000000005515 30789480

[5] Magge SN Westerveld M Pruzinsky T . Long-term neuropsychological effects of sagittal craniosynostosis on child development. J Craniofac Surg 2002;13:99–104 11887004 10.1097/00001665-200201000-00023

[6] Wong KW Forrest CR Goodacre TE . Measuring outcomes in craniofacial and pediatric plastic surgery. Clin Plast Surg 2013;40:305–312 23506771 10.1016/j.cps.2012.11.005

[7] Whitaker LA Bartlett SP Schut L . Craniosynostosis: an analysis of the timing, treatment, and complications in 164 consecutive patients. Plast Reconstr Surg 1987;80:195–212 3602170

[8] van Veelen ML Eelkman Rooda OH de Jong T . Results of early surgery for sagittal suture synostosis: long-term follow-up and the occurrence of raised intracranial pressure. Childs Nerv Syst 2013;29:997–1005 23334575 10.1007/s00381-013-2024-9

[9] Bendon CL Johnson HP Judge AD . The aesthetic outcome of surgical correction for sagittal synostosis can be reliably scored by a novel method of preoperative and postoperative visual assessment. Plast Reconstr Surg 2014;134:775e–786ee 10.1097/PRS.0000000000000633 25347653

[10] Ou Yang O Marucci DD Gates RJ . Analysis of the cephalometric changes in the first 3 months after spring-assisted cranioplasty for scaphocephaly. J Plast Reconstr Aesthet Surg 2017;70:673–685 28262513 10.1016/j.bjps.2016.12.004

[11] Kluba S Rohleder S Wolff M . Parental perception of treatment and medical care in children with craniosynostosis. Int J Oral Maxillofac Surg 2016;45:1341–1346 27117394 10.1016/j.ijom.2016.03.017

[12] Kohan E Wexler A Cahan L . Sagittal synostotic twins: reverse pi procedure for scaphocephaly correction gives superior result compared to endoscopic repair followed by helmet therapy. J Craniofac Surg 2008;19:1453–1458 19098532 10.1097/SCS.0b013e3181897390

[13] van Veelen ML Mihajlovic D Dammers R . Frontobiparietal remodeling with or without a widening bridge for sagittal synostosis: comparison of 2 cohorts for aesthetic and functional outcome. J Neurosurg Pediatr 2015;16:86–93 25910033 10.3171/2014.12.PEDS14260

[14] Guimaraes-Ferreira J Gewalli F David L . Spring-mediated cranioplasty compared with the modified pi-plasty for sagittal synostosis. Scand J Plast Reconstr Surg Hand Surg 2003;37:208–215 14582752 10.1080/02844310310001823

[15] Mutchnick IS Maugans TA . Nonendoscopic, minimally invasive calvarial vault remodeling without postoperative helmeting for sagittal synostosis. J Neurosurg Pediatr 2012;9:222–227 22380948 10.3171/2011.12.PEDS11306

[16] Dalton LJ Kiani S Judge A . Parent and patient-reported outcomes for head shape in children undergoing surgery for single suture synostosis. J Craniofac Surg 2022;33:19–25 34519706 10.1097/SCS.0000000000008117

[17] Pidgeon TE Al Omran Y Farwana R . Outcome measures reported in published clinical research studies in craniosynostosis: a systematic review. J Craniofac Surg 2020;31:1672–1677 32740313 10.1097/SCS.0000000000006680

[18] Millesi M Preischer M Reinprecht A . Do standard surgical techniques lead to satisfying aesthetic results in nonsyndromic sagittal suture synostosis? J Neurosurg Pediatr 2021;28:502–507 34388704 10.3171/2021.4.PEDS2166

[19] Khadka J Gothwal VK McAlinden C . The importance of rating scales in measuring patient-reported outcomes. Health Qual Life Outcomes 2012;10:80 22794788 10.1186/1477-7525-10-80 PMC3503574

[20] Pavri SN Arnaud E Renier D . The posterior coronal incision. J Craniofac Surg 2015;26:243–244 25478982 10.1097/SCS.0000000000001374

[21] Leach P Rutherford S Likhith A . Zig-zag bicoronal scalp incision for cranio-facial cases in paediatric neurosurgery. Childs Nerv Syst 2004;20:483–484 15168055 10.1007/s00381-004-0992-5

[22] Team RDC . R: A language and environment for statistical computing. Vienna, Austria: Foundation for Statistical Computing; V 4.4.0; 2019

[23] Egermark-Eriksson I . Prevalence of headache in Swedish schoolchildren. A questionnaire survey. Acta Paediatr Scand 1982;71:135–140 7136609 10.1111/j.1651-2227.1982.tb09384.x

[24] Fendrich K Vennemann M Pfaffenrath V . Headache prevalence among adolescents—the German DMKG headache study. Cephalalgia 2007;27:347–354 17376112 10.1111/j.1468-2982.2007.01289.x

[25] Gassmann J Morris L Heinrich M . One-year course of paediatric headache in children and adolescents aged 8-15 years. Cephalalgia 2008;28:1154–1162 18727649 10.1111/j.1468-2982.2008.01657.x

[26] Laimi K Metsahonkala L Anttila P . Outcome of headache frequency in adolescence. Cephalalgia 2006;26:604–612 16674770 10.1111/j.1468-2982.2004.01084.x

[27] Petersen S Bergstrom E Brulin C . High prevalence of tiredness and pain in young schoolchildren. Scand J Public Health 2003;31:367–374 14555373 10.1080/14034940210165064

[28] Swain MS Henschke N Kamper SJ . An international survey of pain in adolescents. BMC Public Health 2014;14:447 24885027 10.1186/1471-2458-14-447 PMC4046513

[29] Steenweg-de Graaff J Tiemeier H Steegers-Theunissen RP . Maternal dietary patterns during pregnancy and child internalising and externalising problems. The Generation R Study. Clin Nutr 2014;33:115–121 23541912 10.1016/j.clnu.2013.03.002

[30] van de Beeten SDC Mathijssen IMJ Kamst NW . Headache in postoperative isolated sagittal synostosis. Plast Reconstr Surg 2019;143:798e–805e 10.1097/PRS.0000000000005481 30921138

[31] Lauritzen C Sugawara Y Kocabalkan O . Spring-mediated dynamic craniofacial reshaping. Case report. Scand J Plast Reconstr Surg Hand Surg 1998;32:331–338 9785439 10.1080/02844319850158697

[32] Ridgway EB Berry-Candelario J Grondin RT . The management of sagittal synostosis using endoscopic suturectomy and postoperative helmet therapy. J Neurosurg Pediatr 2011;7:620–626 21631199 10.3171/2011.3.PEDS10418

[33] Nguyen DC Farber SJ Skolnick GB . One hundred consecutive endoscopic repairs of sagittal craniosynostosis: an evolution in care. J Neurosurg Pediatr 2017;20:1–9 28841109 10.3171/2017.5.PEDS16674

[34] Livingston EH Wislar JS . Minimum response rates for survey research. Arch Surg 2012;147:110 22351903 10.1001/archsurg.2011.2169

[35] Longmire NM Wong Riff KWY O’Hara JL . Development of a new module of the FACE-Q for children and young adults with diverse conditions associated with visible and/or functional facial differences. Facial Plast Surg 2017;33:499–508 28962056 10.1055/s-0037-1606361

[36] Kamran R Longmire NM Rae C . Concepts important to patients with facial differences: a qualitative study informing a new module of the FACE-Q for children and young adults. Cleft Palate Craniofac J 2021;58:1020–1031 33153294 10.1177/1055665620969589

