# Supplementary material for: Child-Patient Perspective on Results After Correction of Sagittal Synostosis and the Difference Between Child-Patient and Parent’s Perspectives
Source: J Craniofac Surg. 2024 May 9;35(4):1040–4. doi: 10.1097/SCS.0000000000010263 (PMC11122732; doi:10.1097/SCS.0000000000010263)
Supplement: SUPPLEMENTARY MATERIAL [file scs-35-1040-s001.pdf]

APPENDIX A

Seven questions for children operated for sagittal synostosis

Name: …………………………………………………………………………………………. **Age: …………………. (years)**

1. When I compare the shape of my head to that of others, I find it:

1 ☐	2 ☐	3 ☐	4 ☐	5 ☐
completely similar	similar	a bit different	different	very different

2. I find the visibility of my scar:

1 ☐	2 ☐	3 ☐	4 ☐	5 ☐
Unnoticeable	Barely noticeable	Noticeable	Prominent	Severe

3. Others make remarks about the shape of my head:

1 ☐	2 ☐	3 ☐	4 ☐	5 ☐
never	almost never	sometimes	often	quite often

4. I take into account the shape of my head:

1 ☐	2 ☐	3 ☐	4 ☐	5 ☐
never	rarely	sometimes	often	quite a lot

5. If I could change a part of my head, I would choose: (multiple answers can be chosen)

1 ☐	2 ☐	3 ☐	4 ☐	5 ☐
forehead	sides	back of head	top of head	nothing

6. **I have a headache:**

1 ☐	2 ☐	3 ☐	4 ☐	5 ☐
never	sometimes	few times per month	few times per week	every day

7. **I'm easily distracted:**

1 ☐	2 ☐	3 ☐	4 ☐	5 ☐
not at all	a bit	sometimes	regularly	often
