# Supplementary material for: Child-Patient Perspective on Results After Correction of Sagittal Synostosis and the Difference Between Child-Patient and Parent’s Perspectives
Source: J Craniofac Surg. 2024 May 9;35(4):1040–4. doi: 10.1097/SCS.0000000000010263 (PMC11122732; doi:10.1097/SCS.0000000000010263)
Supplement: SUPPLEMENTARY MATERIAL [file scs-35-1040-s002.docx]

Supplemental Tables

Supplemental Table 1 Attrition analysis

|  | Included  (n = 96) | Excluded  (n = 49) | P – value |
| --- | --- | --- | --- |
| Sex - % (N) |  |  |  |
| Male | 80.2% (77) | 83.7% (41) | 0.778 |
| Female | 19.8% (19) | 16.3% (8) |  |
| Type surgery |  |  |  |
| FBR | 46.9% (45) | 42.9% (21) | 0.777 |
| ESC | 53.1% (51) | 57.1% (28) |  |
| Age at surgery (months) |  |  |  |
| Median (IQR) | 6.0 (4.0 – 11.0) | 5.2 (4.4 – 11.7) | 0.831 |
| Age at time of study |  |  |  |
| Median (IQR) | 11.0 (9.0 – 13.0) | 11.2 (8.0 – 15.0) | 0.900 |
| Cephalic Index |  |  |  |
| Mean (SD)  95% CI | 72.6 (4.23)  71.7 – 73.4 | 72.2 (4.77) | 0.975 |
| Preoperative Cephalic Index |  |  |  |
| Mean (SD)  95% CI | 65.8 (3.82)  64.7 – 66.9 | 66.4 (4.03) | 0.172 |
| Missing | 47 (49.0%) | 26 (53.1%) |  |
| Reintervention - % (N) | 7.29% (7) | 6.12% (3) |  |

Supplemental Table 2 Patient Characteristics

|  | Frontobiparietal Remodeling  (n = 45) | Extended Strip Craniotomy  (n = 51) | Overall  (n = 96) | P – value |
| --- | --- | --- | --- | --- |
| Sex - % (N) |  |  |  |  |
| Male | 77.8% (35) | 82.4% (41) | 80.2% (77) | 0.761^1^ |
| Female | 22.2% (10) | 17.6% (9) | 19.8% (19) |  |
| Age at surgery (months) |  |  |  | < 0.001^2^ |
| Median (IQR) | 11.0 (9.75 – 12.00) | 4.0 (4.0 – 5.0) | 6.0 (4.0 – 11.0) |  |
| Age at questionnaire completion (years) |  |  |  | < 0.001^2^ |
| Median (IQR) | 11.50 (10.0 – 18.0) | 9.0 (8.0 – 11.50) | 11.0 (9.0 – 13.0) |  |
| Cephalic Index |  |  |  | 0.971^3^ |
| Mean (SD)  95% CI | 72.6 (3.83)  71.4 – 73.7 | 72.5 (4.61)  71.2 – 73.9 | 72.6 (4.23)  71.7 – 73.4 |  |
| Preoperative Cephalic Index |  |  |  | 0.303^3CI^ |
| Mean (SD)  95% CI | 66.4 (4.03)  64.6 – 68.1 | 65.2 (3.62)  63.8 – 66.7 | 65.8 (3.82)  64.7 – 66.9 |  |
| Missing | 48.9% (22) | 49.0% (25) | 47 (49.0%) |  |
| Complications - % (N) |  |  |  |  |
| Dura tear | 2.22% (1) | 3.92% (2) | 3.13% (3) | 1.000^1^ |
| Hematoma | 2.08% (2) | 3.92% (2) | 4.17% (4) | 1.000^1^ |
| Reintervention - % (N) |  |  |  |  |
| Parieto-occipital remodeling due to  intracranial hypertension | 8.89% (4) | 1.96% (1) | 5.21% (5) | 0.437^1^ |
| Split skull graft for skull defect | 0.0% (0) | 1.96% (1) | 1.04% (1) | 1.000^1^ |
| Scar correction | 2.22% (1) | 0.0% (0) | 1.04% (1) | 1.000^1^ |

^1^ Chi-squared

^2^Mann-Whitney test

^3^independent t-test

Supplemental Table 3 Patients’ reported outcome measures

|  | **Frontobiparietal Remodeling** | **Extended Strip Craniotomy** | **Overall** | **p-value^1^** |
| --- | --- | --- | --- | --- |
|  | **(n=45)** | **(n=51)** | **(n=96)** |  |
| **Q1: When I compare the shape of my head to that of others, I find it…** | | | | **0.803** |
| Completely similar | 7 (15.9%) | 5 (10.0%) | 12 (12.8%) |  |
| Similar | 13 (29.5%) | 12 (24.0%) | 25 (26.6%) |  |
| A bit different | 18 (40.9%) | 23 (46.0%) | 41 (43.6%) |  |
| Different | 5 (11.4%) | 8 (16.0%) | 13 (13.8%) |  |
| Very different | 1 (2.3%) | 2 (4.0%) | 3 (3.2%) |  |
| Missing | 1 | 1 | 2 |  |
| **Q2: I find my scar…** | | | | **0.315** |
| Unnoticeable | 7 (15.6%) | 14 (28.0%) | 21 (22.1%) |  |
| Barely Noticeable | 18 (40.0%) | 18 (36.0%) | 36 (37.9%) |  |
| Noticeable | 14 (31.1%) | 8 (16.0%) | 22 (23.2%) |  |
| Prominent | 4 (8.9%) | 7 (14.0%) | 11 (11.6%) |  |
| Severe | 2 (4.4%) | 3 (6.0%) | 5 (5.3%) |  |
| Missing | 0 | 1 | 1 |  |
| **Q3: Others make remarks about the shape of my head…** | | | | **0.162** |
| Never | 32 (71.1%) | 40 (78.4%) | 72 (75.0%) |  |
| Almost never | 12 (26.7%) | 7 (13.7%) | 19 (19.8%) |  |
| Sometimes | 1 (2.2%) | 4 (7.8%) | 5 (5.2%) |  |
| Often | 0 (0%) | 0 (0%) | 0 (0%) |  |
| Quite often | 0 (0%) | 0 (0%) | 0 (0%) |  |
| **Q4: I take into account the shape of my head…** | | | | **0.606** |
| Never | 14 (31.8%) | 22 (43.1%) | 36 (37.9%) |  |
| Rarely | 9 (20.5%) | 5 (9.8%) | 14 (14.7%) |  |
| Sometimes | 4 (9.1%) | 4 (7.8%) | 8 (8.4%) |  |
| Often | 7 (15.9%) | 9 (17.6%) | 16 (16.8%) |  |
| Quite a lot | 10 (22.7%) | 11 (22.6%) | 21 (22.1%) |  |
| Missing | 1 | 0 | 1 |  |
| **Q5: If I could change a part of my head, I would choose…** | | | |  |
| Nothing | 34 (75.6%) | 30 (58.8%) | 64 (66.7%) | 0.083 |
| At least one part | 11 (24.4%) | 21 (41.2%) | 32 (33.3%) |  |
| **Q6: I have a headache…** | | | | **0.010** |
| Never | 14 (31.1%) | 6 (11.8%) | 20 (20.8%) |  |
| Sometimes | 21 (46.7%) | 26 (51.0%) | 47 (49.0%) |  |
| Few times per month | 8 (17.8%) | 5 (9.8%) | 13 (13.5%) |  |
| Few times per week | 2 (4.4%) | 13 (25.5%) | 15 (15.6%) |  |
| Everyday | 0 (0%) | 1 (2.0%) | 1 (1.0%) |  |
| **Q7: I’m easily distracted…** | | | | **0.117** |
| Not at all | 7 (15.6%) | 7 (14.0%) | 14 (14.7%) |  |
| A bit | 18 (40.0%) | 14 (28.0%) | 32 (33.7%) |  |
| Sometimes | 6 (13.3%) | 4 (8.0%) | 10 (10.5%) |  |
| Regularly | 10 (22.2%) | 9 (18.0%) | 19 (20.0%) |  |
| Often | 4 (8.9%) | 16 (32.0%) | 19 (20.0%) |  |
| Missing | 0 | 1 | 1 |  |

^1^ Chi-squared

Supplemental Table 4 "If patients wanted to change at least one item of their head, it would be..."

|  | **FBR**  **(n = 11)** | **ESC**  **(n = 21)** | **Overall**  **(n = 34)** | **p-value^1^** |
| --- | --- | --- | --- | --- |
| Forehead | 5 (45.45%) | 1 (4.8%) | 6 (18.8%) | **0.005** |
| Sides | 5 (45.45%) | 5 (23.8%) | 10 (31.2%) | 0.210 |
| Back of head | 1 (9.09%) | 14 (66.7%) | 15 (45.9%) | **0.002** |
| Top of head | 2 (18.18%) | 6 (28.6%) | 8 (25.0%) | 0.519 |

^1^ Chi-squared

Supplemental Table 5 Questionnaire and Age at completion – Spearman correlation

|  | **Age at questionnaire completion** |
| --- | --- |
| Comparison of head shape | -0.075 (P = 0.418) |
| Visibility of scar | 0.264 (P = 0.024) * |
| Remarks from others | 0.097 (P = 0.554) |
| Adaptive behavior | 0.114 (P = 0.180) |
| Change head - nothing | -0.052 (P = 0.616) |
| Headaches | -0.189 (P = 0.020) * |
| Easily distracted | -0.048 (P = 0.479) |

* p < 0.05

Supplemental Table 6 Correlation Aesthetic Questions – Spearman correlation

|  | Adaptive behavior |
| --- | --- |
| Comparison of head shape | 0.179 (P = 0.085) |
| Visibility of scar | -0.032 (P = 0.760) |
| Remarks from others | 0.296 (P = 0.004) * |

* p < 0.05

Supplemental Table 7 Correlation Questionnaire and change in Cephalic Index Child’s and parents questionnaire – Spearman correlation

|  | **Change in Cephalic Index** | |
| --- | --- | --- |
|  | **Child’s questionnaire** | **Parents questionnaire** |
| Comparison of head shape | 0.270 (P = 0.418) | -0.004 (P = 0.777) |
| Visibility of scar | -0.011 (P = 0.024) * | -0.047 (P = 0.768) |
| Remarks from others | 0.172 (P = 0.554) | 0.108 (P = 0.436) |
| Adaptive behavior | 0.122 (P = 0.180) | 0.200 (P = 0.450) |
| Change head - nothing | -0.272 (P = 0.616) | 0.067 (P = 0.794) |
| Headaches | 0.134 (P = 0.020) * | -0.025 (P = 0.822) |
| Easily distracted | 0.017 (P = 0.458) | -0.077 (P =0.517) |

* p < 0.05

Supplemental Table 8 Interrater reliability (Cohen’s Kappa) and percentage agreement between patients and parents per question

|  | **Kappa** | **Z** | **p-value** | **Agreement** |
| --- | --- | --- | --- | --- |
| Comparison of head shape (n = 67) | 0.308 | 3.92 | <0.001 | 44.80% |
| Visibility of scar (n = 69) | 0.321 | 4.19 | <0.001 | 33.3% |
| Remarks from others (n= 69) | 0.095 | 0.972 | 0.331 | 60.9% |
| Adaptive behavior (n = 69) | 0.350 | 3.9 | <0.001 | 47.8% |
| Change head shape (n = 65) |  |  |  |  |
| Nothing | 0.455 | 3.75 | <0.001 | 75.4% |
| Forehead | 0.735 | 6.14 | <0.001 | 96.9% |
| Sides | 0.201 | 1.62 | 0.105 | 90.8% |
| Back | 0.591 | 4.77 | <0.001 | 87.7% |
| Top | 0.175 | 1.45 | 0.147 | 83.1% |
| Headache (n = 69) | 0.449 | 5.82 | <0.001 | 53.6% |
| Easily distracted (n = 65) | 0.499 | 5.82 | <0.001 | 41.5% |

Supplemental Table 9 Correlation age at completion and difference between patients and parent’s questionnaire

|  | **Age at completion** |
| --- | --- |
| Comparison of head shape | -0.004 (P = 0.916) |
| Visibility of scar | -0.034 (P = 0.712) |
| Remarks from others | 0.057 (P = 0.595 |
| Adaptive behavior | -0.037 (P = 0.910) |
| Change head - nothing | -0.091 (P = 0.156) |
| Headaches | 0.066 (P = 0.277) |
| Easily distracted | 0.166 (P = 0.230) |
